# The Premature Senescence in Breast Cancer Treatment Strategy

**DOI:** 10.3390/cancers12071815

**Published:** 2020-07-06

**Authors:** Małgorzata Milczarek

**Affiliations:** Department of Drug Biotechnology and Bioinformatics, National Medicines Institute, 30/34 Chełmska St., 00-725 Warszawa, Poland; m.milczarek@nil.gov.pl

**Keywords:** premature senescence, breast cancer, SASP

## Abstract

Cellular senescence is a permanent blockade of cell proliferation. In response to therapy-induced stress, cancer cells undergo apoptosis or premature senescence. In apoptosis-resistant cancer cells or at lower doses of anticancer drugs, therapy-induced stress leads to premature senescence. The role of this senescence in cancer treatment is discussable. First of all, the senescent cells lose the ability to proliferate, migrate, and invade. In addition, the senescent cells secrete a set of proteins (inflammatory cytokines, chemokines, growth factors) known as the senescence-associated secretory phenotype (SASP), which influences non-senescent normal cells and non-senescent cancer cells in the tumor microenvironment and triggers tumor promotion and recurrence. Recently, many studies have examined senescence induction through breast cancer therapy and potentially using this phenomenon to treat this cancer. This review summarizes the recent in vitro, in vivo, and clinical studies investigating senescence in breast cancer treatments. Senescence inductors, senolytics, as well as their action mechanism are discussed herein. Potential SASP-modulating treatment strategies are also described.

## 1. Introduction

### 1.1. Breast Cancer Treatment Strategy

Worldwide, breast cancer is the most commonly diagnosed type of malignancy in females. In the recently published statistical analyses, it is quoted as the first or second (depending on the country) reason for cancer mortality [1].

Breast cancer is a multifaceted and complex disease. A commonly exploited subtype classification of breast cancer was based on gene expression analysis. It includes luminal A, luminal B, HER2-enriched, and basal-like subtypes [2,3]. In clinical practice, classification into subtypes relies on the levels of traditional biomarker expression, such as: estrogen receptor (ER), progesterone receptor (PR), and epidermal growth factor receptor 2 (HER2), as well as additional markers, i.e., proliferation factor (Ki67), epidermal growth factor receptor (EGFR), and cytokeratin 5/6 (CK5/6) (summarized in Table 1) [4].

The subtype of breast cancer, morphology, size of the tumor, classification grade, as well as the presence of lymph node metastases, are the main factors facilitating the choice of the therapeutic approach. The essential element of the oncologic strategy is the eradication of the tumor via excision of the whole breast (mastectomy) or its part (lumpectomy). Radiotherapy is usually administered after surgery to reduce cancer recurrence and cancer-specific death [5]. The effect of surgical resection can also be enhanced via neoadjuvant or adjuvant systemic therapy. This includes hormonotherapy, chemotherapy, and targeted therapy [6].

Endocrinal therapy (e.g., tamoxifen, letrozole,) is applied in luminal A and B subtypes, based on their sensitivity and dependence on the hormone. In all cases of HER2-positive cancers, humanized monoclonal antibodies (e.g., trastuzumab) and small kinase inhibitors (e.g., afatinib, lapatinib) are used. The majority of patients can also receive widely exploited chemotherapy drugs (e.g., doxorubicin, paclitaxel, cyclophosphamide, carboplatin), except for patients with luminal A subtype. To decrease side effects and improve treatment effectiveness usually a combination of pharmacotherapy is used. Expected outcomes of therapy are malignant cell death, mainly via apoptosis. However, recently other mechanisms of anticancer drug activity such as induction of autophagy or premature senescence are implicated in malignant cell death [7].

### 1.2. Cellular Senescence

Senescence is derived from the Latin word “senex”, meaning old man or old age. The term “senescent cells” means that cells do not divide (i.e., proliferate), however, they remain viable and metabolically active. 

Generally, senescence is classified in two groups: not connected with DNA damage and connected with DNA damage. The 1st group comprises both developmentally programmed senescence and physiological senescence. Developmentally programmed senescence concerns embryonic development of structures and organs. This type of senescence is vital for the development of gall bladder, placenta, gut endoderm, chondroblasts, osteoblasts, etc. On the other hand, the physiological type of senescence is engaged in some processes, e.g., normal megakaryocyte and placental syncytiotrophoblast maturation [8].

The group related to DNA damage is better characterized and was investigated first. In the 1960s, Hayflick found that cultured human diploid cells exhibited a limited time of the number of divisions. He observed increased generation time, gradual cessation of mitotic activity, accumulation of cellular debris, and in the end, total degeneration of the cell culture. Hayflick explained this phenomenon by expression of aging or senescence at the cellular level [9]. In 1990, in *Nature* Harley indicated the dependence of this phenomenon on the amount and length of telomeric DNA. He also pointed out that in cancer cells the activity of telomerase is not associated with telomeres’ length and they do not undergo senescence in long-time cell culture [10]. This type of senescence was named replicative senescence. Some researchers classify it as intrinsic senescence in opposition to extrinsic senescence, which is also known as telomerase independent senescence. Other extrinsic senescence is termed premature senescence and categorized into types such as oncogene-induced senescence, tumor suppressor loss-induced senescence, and therapy–induced senescence. Oncogene-induced senescence is the effect of oncogene activation. Inactivation of the tumor suppressor, e.g., phosphatase and tensin homolog deleted on chromosome 10 (PTEN), leads to tumor suppressor loss-induced senescence. Both types of senescence protect cells from neoplastic growth. Transformation of the early neoplastic cells to fully malignant cells is impaired. Therapy-induced senescence is the effect of chemotherapy treatment or radiation procedure [11]. Those senescences inductors trigger the DNA-damage response (DDR). The cellular kinases that are key mediators of DDR involved in the process include ATM, ATR, and CHK1. ATM and ATR kinases phosphorylate downstream CHK1 and CHK2 kinases. The latter activate senescence signaling pathways by phosphorylation of selected proteins. It is implicated that there exist two main pathways: p53/p21 and p16 and pRB signaling pathways [12].

DNA damage is crucial for senescent cells to secrete a set of proteins with pleiotropic activity such as interleukins, cytokines, chemokines, proteases, growth factors, etc. This capability is known as the senescence-associated secretory phenotype (SASP). SASP activity can be beneficial or deleterious for the organism. The most promising features of SASP (especially inflamtory cytokines and chemokines) constitute the recruitment of immune cells (NK cells, neutrophils, macrophages) and clearance elicitation of senescent cells, e.g., senescent cancer cells. Moreover, initially, non-senescent tumor cells can undergo senescence through SASP paracrine activity and can be removed by immune cells. Immune system activation by senescent cells is also one of the mechanisms engaged in tissue regeneration, wound healing, and attenuation of liver fibrosis. On the other hand, SASP of senescent stromal cells and senescenct cancer cells (both in the later stages of tumor development) creates an immunosupresive environment and promotes tumor gowth, invasion, and metastasis. Futhermore, age-related accumulation of SASP and decline in overall immune function supports tumor development from initial to aggressive and metastatic stages. The detrimental role of senescence in non-maligamny diseases was also proved, e.g., in cataracts, radiation-induced oral mucositis, obesity, sarcopenia, and pulmonary fibrosis [8,13,14].

Furthermore, senescent cells are large, flattened, include more cytoplasmic granularity, and in many cases are multinucleated. Based on these features and secretion of SASP, markers of senescence are proposed (Table 2) [13,15,16].

## 2. Senescence and Anticancer Strategy

Most of the treatment approaches for breast cancer induce senescence, especially in the smallest doses. Higher doses of drugs trigger mainly cell death apoptosis. It should be emphasized that induction of premature senescence in tumor cells means only growth arrest of these cells and could be connected with disease stabilization. Only when senescent cells are cleared by immune system cells can it be regarded as a regression of illness. Furthermore, in some cases, when cancer cell exposure to the inductor of this process is cancelled, senescent cells can escape growth arrests to re-enter the cell cycle [19,20]. Milanovic et al. showed that these cells have a more aggressive growth phenotype. They observed that post-senescent cancer cells exerted higher potency to tumor initiation than cancer cells which have never been senescent. This was a result of the acquisition of stemness-related properties in a cell-autonomous manner during senescence-associated reprogramming. Milanovic et al. studied chemotherapy-induced senescence. In this process, the non-stem bulk leukaemia cells are transformed into self-renewing, leukaemia-initiating stem cells. Similar works on breast cancer models of senescence-associated cancer stemness have never been conducted [19].

### 2.1. Chemotherapy and Senescence Induction 

The effect of anticancer chemotherapeutics has been widely investigated in breast cancer cells (summarized in Table 3). In general, the mechanism of senescence induction by those therapeutics relies on damaged DNA.

#### 2.1.1. Topoisomerase Inhibitors 

Doxorubicin is the most commonly applied chemotherapy drug in breast cancer treatment. It is an anthracycline antibiotic that intercalates into DNA and acts as an inhibitor of topoisomerase II. It can also trigger DNA damage via free radical generation [21]. Doxorubicin is widely regarded as a positive control of senescence induction as it initiates this phenomenon at low and medium doses. This capability of doxorubicin has been demonstrated by many researchers, and importantly, in multiple cell lines (MCF-7, MDA-MB-231, ZR-75, T47D, HTB-122, and CRL2324) [8,22,23,24,25,26,27,28,29,30].

The mechanism of doxorubicin-induced senescence in MCF-7 breast cancer cells was first described in by Elmore et al. The drug initiated the senescence via elicitation of breaks in distal chromosomal sequences, which led to substantial telomere-related cytogenetic abnormalities and resulted in a telomere dysfunction, rather than telomere shortening [26]. Jackson et al. revealed changes in the levels of RB (retinoblastoma protein) family proteins (increased level of p130 and decreased level of RB and p107, respectively). It has been demonstrated that p130 was recruited to key promoters regulating cell cycle transitions, histone deacetylation at those promoters, and gene repression, which led to a long-term growth-arrested state. Furthermore, knockdown of p130 was compensated via p107 and knockdown of p107 via RB [27]. Those results were partially confirmed by Huun and co-workers in MCF-7 cells as well as via other authors (in vitro and in vivo models) since only concomitant inactivation of *P53* and *RB* genes led to a decrease of the senescent cells numbers [26,27,28,29]. Studies on p53-mutated MDA-MB-231 and BT-549 cells revealed that doxorubicin-induced senescence led to an increased expression level of p 21 and p16, respectively [25,30].

Etoposide is another inhibitor of DNA topoisomerase II which blocks religation of the cleaved DNA strand and induces single- and double-stranded breaks. Etoposide is a derivate of a podophyllotoxin [31]. Senescence-induction capability of etoposide was widely documented in MCF-7 cells [32,33]. Wang et al. proved that the etoposide-induced senescence process could be enhanced via expression of E1A-like inhibitor of differentiation (EID3) in MCF-7 cells. EID3 is engaged to form the large structural maintenance of the chromosomes 5–6 (SMC5–6) protein complex, which via interaction with Nse3, a member of the melanoma-associated antigen (MAGE) protein family, regulates chromatin in the DNA damage response [34]. Santarosa et al. compared the etoposide effect in *BRCA1*-deficient and *BRCA1*-proficient cells and showed that the senescence is independent of *BRCA1* status [35].

Topoisomerase I (Top1) inhibitors, camptothecin, and its derivatives, irinotecan, and topotecan, were also investigated as senescence inductors. Top1 is involved in relaxing supercoiled DNA during the replication and transcription process. These drugs form covalent complex drug-DNA-Top1, and its accumulation leads to the activation of the DNA damage response [36].

SN-38, an active metabolite of camptothecin, induces senescence after the treatment of MCF-7 cells. It was demonstrated upon treatment by observation of large, flattened SA-β-gal-positive cells, resistant to apoptosis. The mechanism of this activity was not investigated in breast cancer; however, the studies of Shamanna and Opresko revealed some rationale for its consideration, such as camptothecin, and the ability to drive breast cancer cells into senescence in Werner syndrome protein (WRN) dependent manner. WRN is one of five human RecQ helicases, and participates in DNA repair. Shamanna et al. showed that camptothecin attenuated the WRN level via its degradation by a ubiquitin-mediated proteasome pathway in camptothecin-sensitive breast cancer cells MCF-7, T47D, and ZR-75-1 [36]. Opresko showed the presence of senescent as well as apoptotic cells in a population of MCF-7 cells with knockdown of WRN [37]. Other studies confirmed that irinotecan can also induce senescence in MDA-MB-231 and MC-7 cells. The phenomenon was accompanied by elevated levels of markers such as p-ATM, granularity percentage in cells, 53BP1, γH2AX, secretion VEGF, and IL-8 [25].

#### 2.1.2. Antimetabolites 

Methotrexate is an analog of folic acid competitively inhibiting dihydrofolate reductase (DHF). DHF activity is essential for DNA synthesis because it reduces dihydrofolate to tetrahydrofolate, which in turn serves as a one-carbon donor in a critical step in DNA synthesis [38]. Hattangadi et al. showed that methotrexate triggered the senescence phenotype in MCF-7 cells [39]. Bojko et al. quantified the number of foci per cell caused by the two markers of DNA damage—53BP1 and γH2AX—after exposure to methotrexate of MCF-7 and MDA-MB-231cells. They also observed that cells in a population stained by SA-β-gal got flattened and larger, their granularity increased, and the marked-up expression of p53, p21, and γH2AX [25]. 

Another widely tested antimetabolite is a pyrimidine analog 5-fluorouracil. It generates DNA double-strand breaks (DSB) via its metabolite incorporation into DNA, and its anticancer capability also relies on inhibition thymidylate synthase activity [40]. Elevated expression of p53 and p21 in MCF-7 cells by 5-fluorouracil results in positive stain by SA-β-gal, but no change in cell morphology or induction of DNA damage. Moreover in MDA-MB-231, no markers of senescence were noticed after incubation with this drug [25]. Studies conducted on MDA-MB-231 cells in our laboratory confirm those observations [41]. 5-fluorouracil should not be considered as a senescence inductor in breast cancer cells. However, it can be an element of senescence-induced combination treatment, as was shown by Cerrito in the case of 5-fluorouracil combination with vinorelbine [42].

#### 2.1.3. Microtubule Targeting Agents

Paclitaxel (from *Taxus brevifolia*) binds to the β-tubulin and leads to its stabilization. This results in not forming the correct mitotic spindle and the cell cycle is blocked in the G2/M phase.

Paclitaxel initiates senescence in MCF-7 cells via increased p53 expression and a decrease in pRB level [39]. Bojko et al. additionally showed that the drug facilitated an increase in p21 protein expression. They observed the hallmarks of DNA damage. In an experiment in MDA-MB-231 cells, paclitaxel triggered many of senescence hallmarks (elevated expression of the following proteins: p21, p53, γH2AX, increased granularity in the cells, secretion of VEGF). However, the critical features for senescence identification were not observed (positive β-gal staining and morphology changes) [25]. In Cal51, another triple-negative breast cancer (TNBC) cell line, typical markers of senescence were noticed after incubation with paclitaxel. Interestingly, the authors demonstrated that senescent cells produced more extracellular vesicles (EVs) than non-senescent cells. EVs contained not only the components of SASP—in the experiment with the fluorescent analog of paclitaxel, EVs also contained this substance. Additionally, compounds that engage in cell death pathways e.g., ATPase and annexin, were removed from cells by EVs [43]. 

Another group of microtubule agents are microtubule destabilizers, which bind to the β-tubulin and hinder its polymerization through connection to α-tubulin. They block mitosis at the prometaphase. Vinca alkaloids (derived from *Catharanthus roseus* or semisynthetic) such as vinorelbine, vinblastine, vincristine belong in this category [44].

Vinorelbine can drive breast cancer cells into senescence via a decrease of both protein level and mRNA expression of E2F1, as well as a cancerous inhibitor of PP2A (CIP2A). E2F1 is a transcriptional factor of genes associated with cell cycle distribution, apoptosis, autophagy, DNA damage and repair, as well as senescence [45]. E2F1 promotes the expression of CIP2A, which suppresses the senescence process. Vinorelbine inhibited the transcriptional activity of E2F1 via activation of p21 and triggered the dephosphorylation of Rb. As a result, it also hampered CIP2 expression and senescence induction in MCF-7 cells [46]. Duan et al. showed that vinblastine also triggered senescence in MCF-7 cells. However, this effect was inhibited via c-Jun (c-Jun NH2-terminal protein kinase) expression and its interaction with AP-1 (activator or activating protein 1 c-Jun) [47]. AP-1 is known to be a common suppressor of p53 and p21 and this is probably due to the mechanism of antisenescence activity of c-Jun [48]. Senescence-like morphology also was noticed in MCF-7 cells after incubation with vincristine. The cells were large, flattened, and multinucleated, displaying enlarged lysosomal compartments [49].

#### 2.1.4. Platinum-Based Anticancer Drugs

The main action mechanism of these drugs is based on forming covalent adducts with DNA. The ability of cisplatin to initiate senescence was tested in 3D-cultured breast cancer MCF-7 and MDA-MB-231 cells. Cisplatin triggered senescence, increasing the level of the catalytic subunit of polymerase zeta (REV3L), which was dependent on the ATR-Chk1 pathway. Interestingly, senescence was not observed in the 2D model of breast cancer [50]. Hill et al. also confirmed the expression of 21 genes responsible for the resistance of TNBC to cisplatin, which is connected with senescence [51].

### 2.2. Other Drugs

#### 2.2.1. Poly(ADP-Ribose) Polymerase 1 Inhibitors (PARPis)

Poly(ADP-ribose) polymerase 1 is involved in the repair of DNA single-strand breaks. PARPis are active in cells carrying *BRCA1/2* mutations. *BRCA1* and *BRCA2* genes are critical for high-fidelity repair by homologous recombination. Under the concept known as synthetic lethality, PARPis give rise to the accumulation of unrepaired DNA damage and lead to cell death. Olaparib and talazoparib are used to treat breast cancer patients [52]. Olaparib was shown to be an inductor of senescence in the MDA-MD-231 cell line. This was proved by cell cycle blockade at the G2/M phase of the cell cycle, a decrease of DNA synthesis, and a significant increase in expression of the following genes: *p21*, *CHK2*, *IL-6*, *IL-8*, and *BCL-XL*. These results were confirmed in MDA-MB-231 xenograft tumor models [53].

#### 2.2.2. Antiestrogenic Therapy 

Tamoxifen is known to be an estrogen receptor (ER) modulator and acts as a competitive binder to the ER. The formation of the ER and tamoxifen complex leads to inhibition of DNA synthesis and halts pro-estrogenic effects. Tuttle et al. showed that through this mechanism tamoxifen can induce senescence in MCF-7 cells. The repression of ERα signaling receptor triggers YPEL3 (Yippee-like 3) mRNA expression dependent senescence [54]. YPEL3 is recognized as a growth suppressor and an inductor of apoptosis and senescence [55]. The effect of tamoxifen in MCF-7 cells was also tested by Lee et al. [56]. It blocked protein kinase CK2 activity. CK2 is considered to be required for cell viability and cell cycle progression and is overexpressed in cancer cells [57]. Hampering of CK2 leads to ROS production and induction of senescence in the p53/p21-dependent pathway. 

Another agent used in the treatment of ER-positive breast cancer is fulvestrant. It is the ER antagonist that downregulates ER and has no agonist effects. Fulvestrant senescence induction activity was shown in MCF7 and T47D cells; however, the exact mechanism of this phenomenon was not investigated [58]. Wu et al. suggested a DNA damage-independent mechanism, as they did not notice characteristic foci [33].

#### 2.2.3. HER2-Targeted Tyrosine Kinase Inhibitors 

There are two main groups of HER2-targeted anticancer drugs: monoclonal antibodies and tyrosine kinase inhibitors (TKIs). However, only TKIs can induce senescence. The TKIs act intracellularly and compete with ATP to prevent autophosphorylation and downstream signaling events [59,60]. 

McDermott et al. noticed reversible senescence after incubation with lapatinib (reversible TKI)-sensitive HER2 positive cell lines (HCC1419, SKBR3, EFM-192A, and MDA-MB-361). It was shown that irreversible TKI (neratinib)-mediated irreversible senescence. The authors indicated the necessity of a blockade of more than the HER2 signaling pathway alone for senescence induction via TKIs, since HER2-targeted antibodies failed to do so. They proposed that both HER2 and EGFR inhibition are critical for that effect. Moreover, they proved that lapatinib-initiated senescence in p53-null breast cancer cells and restoration of wt p53 function led to cell death after TKI treatment [59].

## 3. Senescence in Clinical Trials or Clinical Practice 

Senescence was observed in breast cancer patients who underwent adjuvant therapy with cyclophosphamide, adriamycin, and 5-fluorouracil (CAF). Positive staining for SA-β-gal was more frequently observed in tumor sections from patients treated with CAF than in those which did not receive adjuvant therapy. No staining was observed in cases of normal tissue [14].

Pro-senescence drugs have not been investigated in clinical trials as a breast cancer treatment. In many cases, the phenomenon of senescence is used to explain some results of clinical trials.

Cerrito et al. showed that vinorelbine and 5-fluorouracil combined (metronomic schedule) treatment can induce senescence, autophagic cell death, and apoptosis, and all those processes can be regarded as an anticancer mechanism in MDA-MB-231 cells. Moreover, they suggested that the induction of senescence and autophagic cell death were especially responsible for the better response of TNBC patients to metronomic therapy than to a standard therapy schedule [42].

Other authors discussed the prognostic role of CIP2A (a negative regulator of senescence) in a cohort of breast cancer tumor samples from patients with advanced disease. The correlation between CIP2A expression and poor prognosis of HER-negative breast cancer patients was demonstrated. Also, the outcomes of chemotherapy of these patients were examined. Group treatment with vinorelbine followed by 5-fluorouracil, epirubicin, and cyclophosphamide (FEC) was characterized by worse overall survival than the group which received docetaxel followed by FEC. The authors summarized that overexpression of CIP2A was responsible for the tumor resistance to senescence-inducting chemotherapy [40].

Gomes et al. highlighted that 3D culture better reflected the outcomes in patients. They stated that senescence induction can be considered rather as a mechanism of cisplatin resistance in breast cancer tumors, rather than a mechanism of cancer suppression [45].

## 4. Senescence Induction as a New Anticancer Strategy 

### 4.1. Inhibitors of Aurora A

The serine-threonine kinases Aurora A is overexpressed in over 90% of all cases of breast cancer. Aurora A is one of the key elements of cell division, proliferation, and invasion. It plays a significant role in mitosis progression, is engaged in centrosome maturation, mitotic entry, and the assembly of the bipolar spindle [62]. Senescence induction through alisertib (inhibitor of Aurora A) was shown in an animal model of TNBC, but in the in vitro model of TNBC, in CAL51 alisertib induced apoptosis [63,64]. However, Wang et al. demonstrated using the same cell line that alisertib induces in CAL51 senescence [65].

### 4.2. Nanoparticle-Based Drug Delivery Systems

To minimalize the adverse effects of chemotherapy and enhance the accumulation in tumor cells, nanoparticle-based drug delivery systems are commonly utilized. One such example involves doxorubicin-loaded micelles composed of dextran and all-trans-retinal. Their anticancer effects depend on the activation of the RAR signaling pathway which leads to the induction of apoptosis and senescence in MCF-7 cells. Interestingly, the attenuation of senescence reduced apoptosis by about 40%. The senescence was accompanied by a p21 mRNA level increase due to RAR-mediated hypomethylation of p21 promoter [66].

### 4.3. Natural Compound-Induced Senescence in Breast Cancer Cells

Phytocompounds were the first compounds used in anticancer treatment. A great number of commonly applied anticancer chemotherapies are based on synthetic analogs of natural anticancer compounds. It should be highlighted that general natural compounds trigger senescence at low concentration (like anticancer therapy), and they mainly lead to apoptosis at high doses. Natural substances are widely investigated as senescence inductors and usually act through mechanisms such as reactive oxygen species production (ROS-induced senescence), DNA damage (DDR-induced senescence), epigenetic modification, or alteration of overexpressed pathways (oncogene-induced senescence) (Table 4).

#### 4.3.1. ROS Production as the Mechanism of Senescence

ROS production plays a vital role in the initiation of cancerogenesis, progression (moderate or sub-lethal doses), and regression of cancers (high doses) [67]. Cancer cells are regarded as more vulnerable to ROS since they feature a high level of oxidative stress. In the group of phytochemicals, the flavonoids are commonly considered to be the most prominent inductor of ROS. However, non-flavonoids also contribute to this. ROS are generated in mitochondria, endoplasmic reticulum (ER), and peroxisomes. In senescence induction, only the first two compartments of the cell play a pivotal role.

In the studies conducted by Chakraborty et al. and He et al., flavonoids cristacarpin (obtained from *Erythrina suberosa*) and oroxin A (isolated from *Oroxylum indicum*) promoted ROS generation and ER stress markers expression, e.g., GRP-78, GRP-94, and PERK in MCF-7 and MDA-MB-231 cells respectively. The activated p38-MAPK pathway led to the upregulation of p21 and p16 and triggered ER-stress mediated senescence [67,68].

ROS production via mitochondria and mitochondrial potential depletion leads to the activation of oxidative stress sensors, which are senescence mediators, p53 and p21. Li et al. showed that bisdemethoxycurcumin (a natural derivative of curcumin) raised the level of p53 phosphorylation and expression of p21 in MCF-7 cells. Additionally, the activation of p16 and its downstream effector Rb was noticed [69]. Activation of both these pathways is typical for ROS-mediated senescence. Coumasterol, a flavonoid derived from *Glycine max*, inhibited protein kinase CK2, which is known to be crucial for proliferation and oncogenesis. Inhibition of CKII led to a decrease in mitochondrial potential in cancer cells [70]. In MCF-7 cells, this stimulated ROS production upregulated the p53/21 pathway and initiated morphology changes typical of senescence [71].

Mitochondrial ROS production plays a significant role in senescence induction through several natural compounds, e.g., polyphenols from artichoke, sulforaphane, resveratrol, diosmin, salinomycin, and annatto-T3 [22,72,73,74,75,76]. Their mechanism of action was described in detail and these compounds are regarded as epigenetic modulators and/or DNA damage inductors. The role of ROS in both processes was well documented. However, this connection was not investigated in-depth during studies of natural senescence inducers in breast cancer cells [77].

In response to ROS stimuli, extracellular signal-regulated kinase (ERK) can be activated [78]. In studies of diosmin in MCF-7 cells and *Rhus coriaria* extract in MDA-MB-231 cells, ERK phosphorylation correlated with both cytostatic autophagy and senescence induction. In cases of diosmin, it was proven that intensive autophagy leads to apoptosis and ineffective autophagy triggers senescence. Studies on *Rhus coriaria* extract demonstrated the dependence of senescence induction on autophagy via activation of p38 or ERK [22,79].

#### 4.3.2. DNA Damage Inductors 

The majority of phytochemicals which triggered oxidative stress also damaged DNA, resulting in premature senescence. Those include diosmin, salinomycin, and *Rhus coriaria* extract [22,79,80,81,82]. Some compounds, such as curcumin and peloruside A, exert genotoxic activity [39,83]. Others, e.g., sulforaphane, induced both genotoxic and oxidative stress [73].

In this section the genotoxic agent is discussed, because the ROS-generated compounds have been described above. Genotoxic activity of curcumin, derived from *Curcuma longa*, relies on mitotic spindle disturbances that lead to DSB. This triggers DDR activation and induction of senescence in the p53/p21-dependent pathway in MCF-7 cells [83]. Mitosis progression is also inhibited via Peleroside A (from marine sponges) due to its microtubule stabilization activity. Peleroside A induced senescence in a p53/p21 and p16/pRb dependent manner [39].

#### 4.3.3. Epigenetic Modulators

Epigenetic alterations are heritable changes in gene expression which do not concern changes in the DNA sequence. The main epigenetic modifications are DNA methylation, histone modifications, chromatin remodeling, and changes in miRNA profiles. They are considered to constitute a chemopreventive and anticancer mechanism, facilitating apoptosis and premature senescence, e.g., via reactivation of silencing suppressor genes. This is possible because a lot of pro-senescence phytochemicals inhibit DNA methyltransferase or histone deacetylase. This leads to demethylation or acetylation of gene promoters crucial for cancer suppression gene expression and triggers its activation, e.g., for cyclin-dependent kinase (e.g., p16 and p21) [84]. As proved by Mileo et al., polyphenols from artichoke (*Cynara scolymus*) exerted epigenetic activity via facilitating DNA hypomethylation and lysine acetylation in MDA-MB-231 cells [72]. Global DNA methylation patterns were also senescence roots after treatment with diosmin in MCF-7 cells [22]. Salinomycin exerted prosenescence activities via histone H3 and H4 hyperacetylation, which led to an increase in p21 expression in MDA-MB-231 cells [82]. Apart from a decrease in DNMT1 and DNMT3A levels, sulforaphane decreased miR-23b, miR-92b, and miR-381 expression, which correlated with the induction of p21-dependent cell cycle arrest in SK-BR-3 cells [73].

Tumor suppressor gene *DLC1* is the next example of a silencing gene that can be reactivated via the epigenetic activity of phytochemicals in breast cancer cells. It acts in the induction of apoptosis, angiogenesis, and migration inhibition. Resveratrol attenuated expression of DNA methyltransferase (in an ROS dependant manner) and upregulated DCL1 expression in MCF-7 cells. Ji et al. demonstrated that DCL1 activity abrogation hampered p38-MAPK, p27, and p21 expression and causes an increase in Rb level. Resveratrol also increased expression of SIRT1, a NAD-dependent histone deacetylase, via DCL1 activation or independently of DCL1. SiRT1 expression influenced the depletion level of the forkhead transcription factor (FoxO3a), which is engaged in resistance to oxidative stress. SiRT1 also activates NF-κB, which stimulates SASP production detected on these cells via IL-6 protein secretion [74].

#### 4.3.4. Hampering Overexpressed Pathways

Phytochemicals can hamper overactivated pathways in breast cancer cells. They attenuate HER-2 expression, e.g., berberine, tocotrienols, and silipide; inhibit the phosphatidylinositol 3-kinase/ protein kinase B (PI3K/Akt) pathway, e.g., beta-naphthoflavone and norcantharidin [79,85,86,87,88]; and decrease BMI1 expression, e.g., timosaponin A-III [89].

The inhibition of HER-2 signal transduction, typical of the senescence mechanism, can stimulate p53/p21 and p16/Rb pathways. Berberine, isolated from *Berberis vulgaris* and its aromatic, synthetic analogs, induces apoptosis as well as senescent-like growth arrest of HER-2 overexpressing SK-BR-3 breast cancer cells. Those processes were associated with a downregulation of HER2 expression and phosphorylation [90]. Similar effects were noticed after tocotrienols (T3) treatments, which are the isomers of vitamin E (extracted from annatto seeds), and after incubations with silipide (a complex of silybin and phosphatidylcholine) in SK-BR-3 cells. Moreover, the pro-senescence activity of both compounds was demonstrated in animal model studies. The phytochemicals had an influence on the slowing down of tumor development, as well as reducing the number and size of mammary tumors of lung metastasis [76,91].

The Akt signaling pathway is upregulated in breast cancer, and this is regarded as a poor prognosis for the high metastatic activity of malignancy, as well as resistance to therapy [90]. Beta-naphthoflavone (BNF) is a synthetic flavonoid, an agonist of the aryl hydrocarbon receptor (AhR). It inhibits the PI3K/Akt pathway in an AhR-dependent manner, which leads to the downregulation of cyclin D1/D3 and CDK4 and cell cycle arrest in the G1 phase in MCF-7 cells. In the same way, BNF activates the mitogen-activated protein kinase/extracellular signal-regulated kinase (MAPK/ERK) pathway. It is known to be responsible for proliferation. However, its prolonged activation can result in overexpression of p21 and senescence induction. Additionally, activation of AhR triggers ubiquitination and degradation of ERα. Interestingly, this study showed that ERα is crucial for p21 expression after incubation with BNF [85].

Norcantharidin is a synthetic analog of cantharidin (isolated from *Mylabris phalerata* Pall. and *M. cichorii* Linn) which was found to promote senescence via inhibition of both Akt and ERK signaling, which triggered activation of p21 and p16 expression in MDA-MB-231. Simultaneous hampering of Akt and ERK pathways is relevant in TNBC cells since crosstalk between both pathways and blockade of one leads to activation of the other [86]. The study also showed secretion of SASP—IL-6, IL-8, and IL-1β, MMP-1, and MMP—in a manner independent of NF-κB. In addition, the mechanism of norcantharidin action was confirmed in the mouse model [81].

Timosaponin A-III (TA-III), a steroidal saponin derived from *Anemarrhena asphodeloides,* influenced senescence induction via downregulation of c-Myc oncoprotein and decreasing expression of BMI1 (B lymphoma Mo-MLV Insertion region 1) in MCF-7 and MDA-MB-231 cells. BMI1 facilitates proliferation, migration, invasion of cancer cells, and breast cancer cell stemness. An additional activity of TA-III comprises upregulation of miR200c and miR-141, which exerts the second means of BMI1 inhibition [87].

The prediction of the SASP’s role in therapy is difficult because the composition of this protein mixture is dependent on senescence stages, type of senescence inductor, and tumor localization. The most described SASP of breast cancer senescent cells is the SASP produced after doxorubicin treatment (Table 5).

Chemokines and cytokines can influence different types of immune cells, therefore it can have two different effects: it can promote cancer progression through immunosuppression or it can decrease the cancer cell number by their clearance [18,24,86,93]. Matrix enzymes can remodel the microenvironment to promote cancer initiation and development. Some SASP proteins, e.g., cytokines and THBS1, act in maintenance of senescence [23,29,30,86,94].

## 5. Prevention of SASP in Breast Cancer Studies

Primary evidence stated that senescence as a tumor-suppressor mechanism, especially in cancer cells that are resistant to apoptosis, was favorable. Additionally, lower doses of drugs are required to induce senescence, which leads to the reduction of the adverse effects of chemotherapy. However, the SASP phenomenon is deleterious through a bystander effect on normal and cancer cells. This can promote cancer relapse and chemotherapy side effects, e.g., bone marrow suppression and cardiac dysfunction [102]. Therefore, there are three approaches concerning the SASP in cancer treatment: firstly, prevention of the initiation of senescence; secondly, amelioration of the senescence bystander effect by senostatics, and thirdly, the elimination of senescent cells by senolytics.

### 5.1. Senostatics and Breast Cancer Studies

The increased senescence bystander effect relies on SASP activity or its component modification. In both cases, a pivotal role is played by alteration of activity key regulators of SASP—p38MAPK and NF-κB. In studies on breast cancer cells, ginsenoside Rh2, apigenin, and trabectedin were tested as senostatics [23,94,103].

The study of MDA-MB-231 breast cancer cells and MCF-10A normal breast cells demonstrates that ginsenoside Rh2 (from *Panax ginseng*) restores the secretory production and reduces the doxorubicin-induced bystander effect. The SASP of MDA-MB-231 produced after incubation with this anticancer drug triggers cell cycle arrest in MCF-10A cells and SASP secreted by both types of cells stimulates proliferation as well as invasion and migration of non-senescent cancer cells. Treatment with Rh2 does not decrease the number of senescent cells, but suppresses SASP activity through the inactivation of p38-MAPK and NF-κB pathways [94]. 

The influence on SASP by apigenins (found in many plants, e.g., *Acacia arabica)* was tested in IR-induced senescent HCA2 fibroblasts. The senescent cells were incubated with the vehicle or with the vehicle containing apigenin. Next, the collected conditioned media were added to aggressive MDA-MB231 and non-aggressive ZR75.1 breast cancer cells. The vehicle of non-apigenin-incubated cells stimulated the proliferation of both tested cancer cell lines more than the vehicle of apigenin-incubated cells. Apigenin ameliorated the ability of the SASP by suppressing the activity of p38-MAPK and NF-κB [103].

Trabectedin (isolated from the tunicate *Ecteinascidia turbinate*) is another modulator of SASP. It is a natural potential anticancer agent. The influence of trabectedin on senescent breast cancer cells was tested after the treatment of MCF-7 cells with doxorubicin. The results mainly rely on the modulation of NF-κB transcriptional activity, especially by suppression of the RelA/p65 subunit of NF-κB. At lower doses, it influenced the components of SASP. Trabectedin reduced expression of proinflammatory cytokines (IL-6, IL-8, TNF-alpha). Interestingly, the expression level of chemoattractant of monocyte and T-cell CXCL10 rose. Additionally, trabectedin positively influenced the recruitment of innate immune cells [23].

### 5.2. Senolytics and Breast Cancer Studies

**Figure 1:**
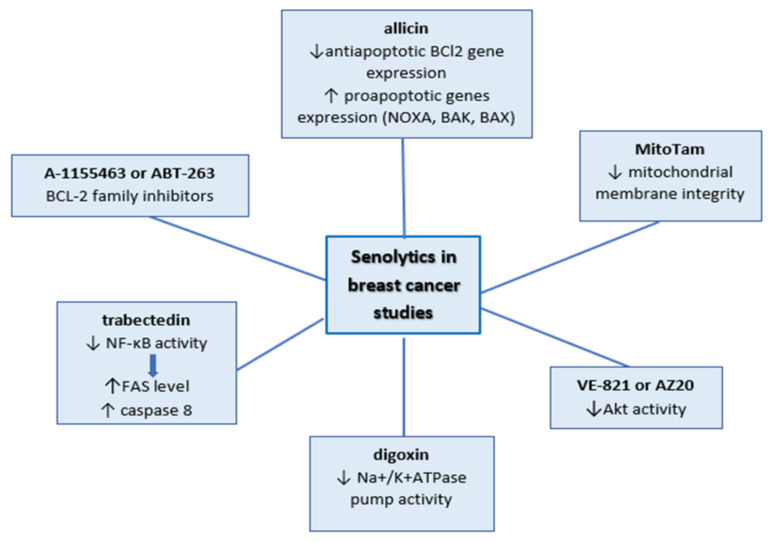
Senolytics tested in breast cancer studies.

Senolytics are compounds selectively killing the senescent cell. The group of Zhu, Y. et al. was the first research group to make a hypothesis that senescent cells developed antiapoptotic pathways. They explored the hypothesis using the bioinformatic approach and small interfering RNA (siRNA) technology to identify the targets of these compounds. Even though the authors studied senescence as a factor of chronic diseases, their results are significant and valid in the case of senescent cells in cancer [25]. Zhu et al. and Fuhrmann-Stroissnigg et al. defined targets of senolytics studying senescent-cell anti-apoptotic pathway elements such as anti-apoptotic proteins BCL-2 (BCL- W and BCL- XL), the transcription factors p53 and p21, hypoxia-inducible factor 1 (HIF1α), phosphatidylinositol-4,5-bisphosphate 3-kinase (PI3K) and protein kinase B (PKB; also known as Akt), the serine protein inhibitors (serpins), and the heat shock protein HSP90 [104,105]. Senolytics tested in breast cancer studies are summarized in Figure 1.

Studies conducted on senescent breast cancer cells involved only a few of those factors, along with the large group of BCL-2 family inhibitors. BCL-2 family inhibitors (A-1155463 or ABT-263) were tested in olaparib-induced senescence in breast cancer xenograft models. The experiments showed that combination treatment enhances cell death in comparison with treatments by separate agents [53]. ATB-263 senolytic activity was also investigated in a p16-3MR mouse model that was injected with MMTV-PyMT breast cancer cells. Following the surgical removal of the tumor, the mice were treated with doxorubicin and ABT-263. The use of this model allowed the detection of p16-positive senescent cells in a living mouse. Elimination of the senescent cells via ABT-263 hampered tumor recurrence and metastasis. Furthermore, it was shown that the deletion of the senescent normal host cells was essential to achieve this result [102].

In recent studies, allicin (garlic compounds) showed senolytic activity in doxorubicin-treated MCF-7 and HCC-70 cells. Even though the mechanism of this phenomenon has not been studied, it would appear to be the effect of downregulation of antiapoptotic *BCl2* gene expression and upregulation of proapoptotic genes (*NOXA, BAK, BAX*) [106].

At the higher doses, trabectedin decreases the viability of senescent cells by triggering apoptosis via downregulation activity of NF- κB. This led to an increase in FAS level and activation of caspase 8, as well as the induction of mitochondrial membrane depolarization [23].

Gomes et al. showed another way to decrease the number of senescent cells. They proposed the modification of the signal pathway via inhibition of Akt activity during cisplatin treatment. The 3D-cultured MCF-7 cells were co-treated with VE-821 or AZ20 (structurally different inhibitors of Akt) and cisplatin. They observed a significant decrease in cell viability after combination treatment in comparison to agent treatment alone [50].

The most recent proposition of specific elimination of senescent cells is mitochondria-targeted tamoxifen (MitoTam), which will be tested on TNBC patients in clinical trials in the near future. In vitro studies showed that MitoTam decreased the majority of the doxorubicin-induced senescent cells on 4T1 and MCF7 cells. MitoTam led to the loss of mitochondrial membrane integrity. The authors concluded that the low level of adenine nucleotide translocase-2 (ANT2) is crucial for its activity in senescent cells [107].

Senescent cells are also characterized by slight depolarization of the plasma membrane, which can be enhanced via some senolytics such as digoxin (cardiac glycosides). It disturbs the Na+/K+ATPase pump activity via binding to its alpha subunit and leads to a drop in the intake of K+ and increases the release of Na+ out of cells. Triana-Martínez et al. proved this senolytic activity in injected PDX375 breast tumors in nude mice treated with the combination of doxorubicin and digoxin. The combined treatment result was more prominent than treatment with these drugs alone [108].

Inao et al. showed that the combination of chemotherapy with anticancer immunotherapy increases the efficacy of treatments via the elimination of senescent cells. The cytotoxic effect of anti-EGFR chimeric antigen receptor (CAR)-T cells grew after incubation of TNBC cells with doxorubicin. The authors previously showed that doxorubicin induces senescence, and they observed SASP in MDA-MB-231 and BT-549 cells. They noted more apoptotic cells after combined treatment, compared with other treatments. Their explanation of this phenomenon was that SASP components activate immune cell-mediated cytotoxicity [30].

## 6. Conclusions

Premature senescence is an inherent outcome of breast cancer treatment strategy, as well as an element of novel strategies that rely on applying phytochemicals or targeted therapy. In the beginning, premature senescence was regarded as a promising cytostatic therapy for apoptosis-resistant cancers. However, SASP analysis showed that senescence can promote cancer development, enhance metastasis, impair immunosurveillance, and generate other detrimental results. Recently, some authors demonstrated that senescence mediated adverse effects of chemotherapy. Therefore, studies on senolytics or senostatics agents, as well as on senescent cell biology are being conducted.

Currently, combined cancer treatments inducing senescence as an anticancer mechanism are being tested in cancer clinical trials. Those combinations contain compounds which are known to attenuate SASP’s negative influence. However, this effect has not been investigated in the case of breast cancer. Further developments in this strategy for breast cancer treatment could be regarded as a milestone for breast cancer therapy.

## Figures and Tables

**Table 1:** Breast cancer classification, characterization, and model cell lines used in cancer treatment studies.

Subtype	Immunochemistry Markers	Systemic Therapy	Overview
luminal A	ER+ and/or PR+, HER2− and low Ki67	hormonal therapy	The most common subtype (50–60% of all cases) The relapse rate is the lowest in comparison to other types The recurrence is mainly in the bone Good prognosis for the patient Model cell lines: MCF-7, T47D
luminal B	HER negative: ER+ and/or PR+ or high Ki-67	hormonal therapy + chemotherapy	15–25% of breast cancer patients Relapse and recurrence occur more often than in the luminal B subtype Model cell lines: ZR-75
	HER positive: ER+ and any PR−, any Ki-67	hormonal therapy +anty-HER therapy + chemotherapy	
HER2-enriched	ER−, PR−, HER2+	anty-HER therapy (humanized monoclonal antibodies and small kinase inhibitors ) + chemotherapy	20–30% of all cases of breast cancer Aggressive biological and clinical behavior Model cell lines: SK-BR-3, TUBO, EFM-192A, MDA-MB-361
basal-like	ER−, PR−, HER2−, CK5/6+, and/or EGFR+	chemotherapy	80% of basal-like subtype is triple-negative 15–20% of all cases of breast cancer Aggressive clinical behavior High rate of metastasis to the brain and lung Poor patient prognosis Model cell lines: MDA-MB-231,BT-549 (HTB-122),Crl-2324, Cal51

**Table 2:** Main changes of cell morphology, markers, and methods in senescence process investigation.

Change	Markers	Methods
increased lysosomal content	↑senescence-associated beta-galactosidase (SA-β-gal) ↑lipofuscin	x-gal biochemical assay cytometric quantification of C12FDG fluorogenic β-galactosidase substrate Sudan Black B staining
permanent cell cycle arrest	↑cell cycle inhibitors: p21, p16,p19	mRNA level and protein level determination methods
DNA damage	↑γH2AX (a phosphorylated form of the histone variant H2AX) and 53BP1 (p53 binding protein 1) ↑ phosphorylated p53	immunostaining/immunoblotting
loss of the proliferation activity	↓Ki67 protein or 5-bromodeoxyuridine incorporation (expect the case of polyploidization during senescence [8])	immunostaining
loss of the nuclear envelope integrity	↓LaminB1 [17] ↑cytoplasmic chromatin fragments (CCFs) [18]	mRNA level and protein level determination methods DAPI staining
senescence-associated heterochromatin foci (SAHF)	↑ the histone H2A variant macroH2A, ↑heterochromatin protein 1 (HP1) proteins ↑ lysine 9 di-or tri-methylated histone H3 (H3K9Me2/3)	immunofluorescence staining
SASP	↑interleukins e.g. IL-6, IL-1a, IL-1b ↑chemokines e.g. IL-8, ↑cytokines such as colony-stimulating factors (CSFs) e.g. GM-CSF, ↑growth factor expression e.g., vascular epithelial growth factor (VEGF) ↑proteases such as metalloproteinases e.g.,MMP-1 and MMP-3, collagenase-1 (MMP-1) [13]	ELISA in culture medium

**Table 3:** Senescence induction via standard breast cancer therapy.

Drug	Cell line, (Concentrations)	Senescence Markers and Mechanism
doxorubicin	MCF-7, MDA-MB-231 (100 nM)	positive SA-β-gal staining, characteristic morphology, increased percentage of granular cells, γH2AX and 53BP1 foci, increased level of p-ATM and p21expression SASP: IL-8, VEGFR [25]
	MCF-7 (1 μM)	positive SA-β-gal staining, p53/p21 pathway activation, telomere-related cytogenetic abnormalities induction [26]
	MCF-7, ZR-75 (1 μM)	p53/p21 pathway activation, G1 and G2 cell cycle block, increased p130 and decreased RB and p107 protein expression [27]
	MCF-7, T47D, HTB-122, CRL2324 (0.5 and 1 μM)	positive SA-β-gal staining, concomitant inactivation of P53 and RB genes lead to inhibition senescence [28]
	MCF-7, ZR-75.1 cells and a different status of p53 MMTV-Wnt1 mice	positive SA-β-gal staining, p53, and p21 pathway dependence ceased incorporating BrdU, characteristic morphology, phosphorylation of STAT3, SASP: IFNg, IL-6, CXCL2, TNFa, CXCR2, CXCL1 [29]
	MDA-MB-231 (250 nM), BT-549 (100 nM), MCF-7 (200 nM)	positive SA-β-gal staining, γH2AX expression, elevated p21 or p16 expression, SASP: IL-6, IL-8 (only MDA-MB-231, BT-549) [30]
etoposide	MCF-7 (2 μM)	positive SA-β-gal staining, morphology, G1 cell cycle phase block [32]
	MCF-7 p21 (with ectopic expression of p21) (10 μM)	raised formation 53BP1 foci (24h), increased activity of SA-β-gal (5 days) [33]
	*BRCA1*-deficient (HBL100-, MCF7-, and T47D-derived clones with a silenced *BRCA1*) and *BRCA1*-proficient cells (HBL100, MCF7, and T47D) (2.5 and 5 μM)	the activity of SA-β-gal independent on *BRCA1* [35]
SN-38	MCF-7 (100 ng/mL)	large, flatted, resistant to apoptosis and SA-β-gal-positive cells [32]
camptothecin	MCF-7, T47D, ZR-75-1 (10 μM)	positive SA-β-gal staining, the decrease in WRN expression enhanced senescence intensity [36]
irinotecan	MDA-MB-231, MC-7 (5 μM)	positive SA-β-gal staining, elevation level of following markers: p-ATM, percent of granularity in cells, increased in 53BP1 and γH2AX and secretion SASP: VEGF and IL-8 [25]
methotrexate	MCF-7 (10 μM)	positive SA-β-gal staining, p53 and p21 [61]
	MCF-7 (2.5 μM) MDA-Mb-231 (30 μM)	53BP1 and γH2AX foci observation, cells stained via SA-β-gal, many flatted and large cells, granularity increased, expression of p53 (phosphorylated and not unphosphorylated forms), p21, γH2AX grew, SASP: VEGF and IL-8 [25]
paclitaxel	MCF-7 (3.3 nM)	positive SA-β-gal staining, elevation of p53 expression levels, decrease in pRB level [39]
	MCF-7 (56 nM) MDA-MB-231 (5μM)	positive SA-β-gal staining, flatted and large cells elevation of p21expresssion, γH2AX, and 53BP1 foci observation elevated expression of the following proteins: p21,p53, γH2AX, increased % of granularity in the cells, SASP: VEGF [25]
	MDA-MB-231, Cal51 (75 nM)	positive SA-β-gal staining, G2/M cell cycle block, increased of p21 and p16 expression, intensive production of EV contains drug [43]
vinorelbine	MCF-7 (20 nM and 30 nM)	positive SA-β-gal staining, flattened cellular morphology, increase in p21 expression, inhibition of E2F1 and CIP2A protein expression [46]
vinblastine	MCF-7 (0.3 μM)	positive SA-β-gal staining, decrease in c-Jun expression, drop of AP-1 activation [47]
vincristine	MCF-7 (0.3 μM)	large, flatted and multinucleated cells, G2/M cell cycle block, an increase in the size of individual lysosomes and the total volume of the lysosomal compartment [49]
cisplatin	MDA-MB-231, MCF-7 (60 μM)	positive SA-β-gal staining, the rise in γ-H2AX level and the mRNA expression level of p21, activation ATR-Chk1 pathway via elevated REV3L expression [50]
olaparib	MDA-MD-231 (2.5 µM)	positive SA-β-gal staining, G2/M phase cell cycle block, decrease in DNA synthesis, drop in expression of the following genes: p21, CHK2, IL-6, IL-8, and BCL-XL [53]
tamoxifen	MCF-7 (0.5 μM)	positive SA-β-gal staining, YPEL3 expression dependent senescence [54]
	MCF-7 (5 and 10μM)	positive SA-β-gal staining, decrease CK2 activity, ROS production, activation p53–p21Cip1/WAF dependent pathway [56]
fulvestrant	MCF7, T47D (5, 10, 50 µM)	positive SA-β-gal staining, reduction in both ERα and MDM2 [58]
lapatinib neratinib	SKBR3 (250 nM), HCC1419 (250 nM), EFM-192A (250 nM), MDA-MB-361 (500 nM), MDA-MB-453 (1 µM) MCF7 (1 µM)	positive SA-β-gal staining, increase in p15, p27 expression [59]

**Table 4:** Senescence induction via phytochemicals.

Natural Compounds	Cell Lines or Other Models, (Concentration)	Detection and Effects
**I. Flavonoids**		
diosmin	MDA-MB-231, MCF-7, SK-BR-3, (5 and 10 µM)	positive SA-β-gal staining, an increase in the levels of p53, p27, p21, G2/M phase of cell cycle arrest, ROS production, cytostatic autophagy accompanied SISP, only in MDA-MB-231 and SK-BR-3 cells: ERK1/2 activation only in MCF-7 cells epigenetic changes (hypomethylating agent), DSBs, SSBs (single-strand breaks) [22]
silybin (silymarin flavonoids) complex with phosphatidylcholine	SK-BR-3 (63.2, and 126.5 mg/mL), mouse models for HER2-overexpressed breast cancer (414 µmol/L/kg silybin)	positive SA-β-gal staining, cells with enlarged and flattened morphology, down-regulation of HER-2/neu expression, an increase in expression of p53 mRNA, additional in mammary tumors: the boost of the number of neutrophils, CD4, and CD8 lymphocytes, reduction of the average mean tumor number, decreasing the percentage of mice with metastasis [91]
oroxin A	MDA-MB-231 (5 ÷ 20 µM)	positive SA-β-gal staining, SAHF, G2/M phase of cell cycle arrest, increased expression of p21 (both protein and mRNA), reorganization of microtubules and actin cytoskeleton, ROS production, endoplasmic reticulum (ER) stress-mediated senescence expression of ER stress markers (ATF4 and GRP78), elevated phosphorylation of p38 [67]
betanaphthoflavone	MCF-7 (10 µM)	positive SA-β-gal staining, G0/G1 phase of cell cycle arrest, downregulation of cyclin D1/D3, CDK4, increased in the expression of p21, activation MAPK-ERK signaling, AhR-dependent inhibition of the PI3K/Akt pathway [85]
cristacarpin	MDA-MB-231 (1, 5, 10 µM) 4T1 cells implanted into Balb/c mice (950 mg/kg/body weight)	positive SA-β-gal staining, cells with enlarged and flattened morphology, SAHF, G0/G1 phase of cell cycle arrest, p21upregulation, ROS production, decrease in the expression of Cdk-2, cyclinD1, activation MAP kinase pathway, ER stress (amplification of expression markers viz. GRP-78, GRP-94, and PERK) positive SA-β-gal staining, inhibition growth and development of tumor [68]
coumestrol	MCF-7 (10 ÷ 50 µM)	positive SA-β-gal staining, activation of the p53-p21 pathway, ROS production, inhibition of CKII [71]
**II. Non-flavonoids**		
curcumin	MCF-7 (10 and 15 µM)	positive SA-β-gal staining, mitotic arrest, the lack of BrdU incorporation, 53BP1 and γH2AX foci, increase in the level of γH2AX, p53 and p21 proteins, the structure of mitotic spindle disturbances [83]
bisdemethoxycurcumin	MCF-7 (20 µM)	ROS production, activation of p53/p21 and p16/Rb pathways, a reduction of mG2/M phase of cell cycle arrest [69]
peloruside A	MCF-7 (9.6 nM)	positive SA-β-gal staining, G0/G1 phase of cell cycle arrest, elevated expression of p53 [39]
polyphenols extracted from artichoke	MDA-MB231 (30 µM)	positive SA-β-gal staining, upregulation of p16, p21, ROS production, epigenetic alterations (modulating DNA hypomethylation and increase of lysine acetylation) [72]
norcantharidin	MDA-MB-231 (21.83 µM) MDA-MB-231 implanted into mice model with injected, (28 mg/kg)	positive SA-β-gal staining, decrease in phosphorylation of Akt and ERK1/2, rise in p21 p16 level and γ-H2AX expression level, G2/M phase of cell cycle arrest, SASP: IL-6, IL-8 and IL-1β, MMP-1, MMP-3 Additionally in the mouse model: decrease in tumor volume [81]
timosaponin A-III	MDA-MB-231 and MCF7 (2 and 4 µM)	positive SA-β-gal staining, rise in expression of miR-141 and miR-200c leading to inhibited expression of BMI1, c-Myc downregulation, histone posttranslational modification activity of PRC1 [89]
berberine derivates	SK-BR-3 (50 µM)	a rise in mRNA expression of p53, p21, p16, and PAI-1, a downregulation of HER-2/neu expression [92]
resveratrol	MCF-7, MDA-MB-231 (20 µM)	positive SA-β-gal staining, rise in expression of p38MAPK, p27, p21, downregulation of Rb and p-Rb protein, ROS production, a reduction in the mitochondrial membrane potential, down-regulation of mitochondrial MT-ND1, MT-ND6, and ATPase8 mRNA level, decrease in PGC-1α protein level, downregulation of DNMT1, increase in DLC1 level, SIRT1, NF-κB level, SASP: IL-6, decrease in FoxO3a level [74]
annatto-T3	TUBO, SKBR3 (50 μM) FVB/N mice (100 mg/kg)	positive SA-β-gal staining, ROS production, decrease in the mitochondrial membrane potential, upregulation of p53, p21, and p27 mRNA, reduction in HER-2/neu mRNA expression, in mouse model—decrease in following parameters: tumor development, number and volume of the tumor, size of lung tumor metastasis [76]
sulforaphane	MCF-7, MDA-MB-231, SK-BR-3 (5 ÷ 10 µM)	positive SA-β-gal staining, elevation in p21 and p27 levels, ROS production, DSBs, ATM phosphorylation increase, 53BP1 foci formation, epigenetic modification: DNA hypomethylation, decrease in levels of DNMT1 and DNMT3B, microRNA profile changes, cytostatic autophagy accompanied [73]
salinomycin	MDA-MB-231 (10, 25 µM)	positive SA-β-gal staining, G2/M phase of cell cycle arrest, γH2AX foci observation, upregulation p21, histone H3 and H4 hyperacetylation [82]
*Rhus coriaria* extract	MDA-MB-231 (100–600 µ/mL)	positive SA-β-gal staining, G1 phase of cell cycle arrest upregulation of p21, downregulation of cyclin D1, p27, PCNA, c-Myc, phospho-RB, no proliferative recovery, cytostatic autophagy accompanied [79]

**Table 5:** SASP in doxorubicin-treated senescent cells.

SASP Element	Examples	Effect
cytokines	IL-6 [23,29,30,94] IL-8 17 [25,30,88,94] IL-1α/β [88,94],	proinflammatory cytokines, maintenance of senescence, promotion of tumorigenesis and chemotherapy resistance
	IL-18 [88]	proinflammatory cytokine, induction fibroblast senescence [95]
	TNF-α[23] IFNg [29]	proinflammatory cytokines, inductors of senescence [96]
	IGFBPs, uPA, FGF-6 [94]	invasiveness and metastatic activity of cancer cells
chemokines	CXCL2, CXCR2, CXCL10 [29] CXCL11, CXCL10 [88] CXCL10 [23]	chemoattractant of monocyte and T-cell, therefore it is known as a tumor-suppressive factor [97]
growth factors	VEGFR [25]	induction of vascular permeability during inflammation or inhibit senescence [98,99]
	GM-CSF [94]	inhibitors of antitumor immunity promote tumor progression and metastasis [100]
	TGFβ [88]	promoting tumor progression, including evasion of immune surveillance, autocrine mitogen and cytokine production, epithelial-mesenchymal transition [88]
matrix enzymes	MMP1 [88] MMPs [94]	remodeling microenvironment to promote cancer initiation and development [101]
matricellular protein	THBS1 [88]	maintenance of senescence [88]
